# Case Report: Recurrent coronary in-stent restenosis as the primary manifestation of non−criteria antiphospholipid syndrome confirmed by anti-phosphatidylserine/prothrombin IgM antibodies

**DOI:** 10.3389/fimmu.2026.1852153

**Published:** 2026-06-10

**Authors:** Mingming Tuo, Yunqing Li, Zhangwei Shi, Miao Ma, Yuan Feng, Jin Yu

**Affiliations:** 1 Department of Cardiology, Xijing Hospital, The Fourth Military Medical University, Xi’an, China; 2 The Graduate School of Xi'an Medical University, Xi’an, China; 3 Department of Cardiology, Xi’an No.3 Hospital, Xi’an, China; 4 Department of Immunology, Tangdu Hospital, The Fourth Military Medical University, Xi’an, China

**Keywords:** case report, non‑criteria antiphospholipid syndrome, aPS/PT IgM antibody, in-stent restenosis, immunothrombosis

## Abstract

**Background:**

A 47-year-old female with no traditional CAD risk factors developed recurrent in-stent restenosis (ISR) one year after LAD stenting. She had recurrent pregnancy loss, chronic urticaria, anemia, and proteinuria, with negative conventional antiphospholipid antibodies.

**Case:**

After second stenting, aPS/PT IgM was elevated (51.65 U/mL), supporting the diagnosis of non−criteria APS. Thromboelastography (TEG) showed decreased R time, increased MA and CI, suggestive of a hypercoagulable state. Warfarin (INR 2-3) and hydroxychloroquine (200 mg bid) plus DAPT led to no chest pain recurrence and stent patency at 21-month follow-up.

**Conclusions:**

Recurrent ISR without traditional risk factors should raise suspicion for non−criteria APS. A moderately elevated aPS/PT IgM may serve as a diagnostic clue. Anticoagulation plus immunomodulation may help prevent further thrombotic events.

## 1 Introduction

Antiphospholipid syndrome (APS) is an autoimmune disorder characterized by thrombosis and obstetric morbidity. The 2023 ACR/EULAR classification criteria for APS have refined the diagnostic algorithm, emphasizing persistent positivity for lupus anticoagulant (LA), anticardiolipin (aCL), or anti-β2-glycoprotein I (aβ2GPI) antibodies at medium-to-high titers (1, 2). A subset of patients with typical APS features but persistently negative standard assays is increasingly recognized; for these patients, the term “non−criteria APS” has been proposed, as non−criteria antibodies such as anti−phosphatidylserine/prothrombin (aPS/PT) may be positive (3, 4). IgM isotypes of such antibodies have been particularly implicated in arterial thrombosis.

Although APS infrequently involves the coronary arteries directly, its associated autoantibodies can exacerbate pre-existing local vascular inflammation and thrombosis by activating endothelial cells, promoting platelet aggregation, and inducing fibrin deposition. In non−criteria APS, such antibodies may drive thrombotic events through alternative immunological pathways (5, 6). Non−criteria APS manifesting as recurrent coronary in−stent restenosis is extremely rare and often misdiagnosed as conventional atherosclerosis. We report a case diagnosed by aPS/PT IgM testing, highlighting potential immunopathological mechanisms in unexplained stent restenosis.

## 2 Case description

A 47-year-old woman with no history of hypertension, diabetes, dyslipidemia, or smoking presented in March 2022 with exertional chest pain and dyspnoea. Her past history included penicillin allergy and a father with CAD. She had long-standing multisystem symptoms: chronic urticaria, recurrent oral ulcers, arthralgia, alopecia, persistent anaemia, and proteinuria. Obstetric history: three spontaneous abortions and two second-trimester losses (24 and 28 weeks). This suggested an underlying autoimmune process.

On admission, physical examination was unremarkable. Troponin I was 0.042 ng/mL (<0.03). Coronary CTA showed mid-distal LAD occlusion and severe proximal stenosis, confirmed by angiography (100% stenosis). A drug-eluting stent was placed in the LAD, and drug-eluting balloon dilation of the first diagonal branch with thrombectomy was performed. Dual antiplatelet therapy (DAPT, aspirin+clopidogrel) and intensive lipid-lowering therapy were started, with symptom relief.

In May 2023, while on DAPT, chest pain recurred. CTA revealed in-stent LAD occlusion, confirmed by repeat angiography. Physical examination now showed scattered erythema on trunk and limbs. A tandem stent was implanted in the proximal-to-mid LAD; Intravascular ultrasound (IVUS) confirmed optimal apposition.

Following the second intervention, the patient developed scattered systemic erythema. Initial treatment with intravenous glucocorticoids (dexamethasone 5 mg/day) was ineffective. After excluding contrast allergy, an immune-mediated process was suspected, prompting immunology referral.

Given systemic symptoms, obstetric morbidity, and arterial thrombosis with negative standard serology, non−criteria APS was suspected. The major diagnostic challenge was persistently negative standard aPL tests despite high clinical suspicion, necessitating the use of non-criteria antibody testing. Further workup: (1) Standard antibodies (LA, aCL, aβ2GP1, ANA, anti-dsDNA) and thrombophilia tests were normal; (2) hs-CRP and total IgE were elevated; TEG showed decreased R time, increased MA and CI (acquired hypercoagulable state); (3) Urinalysis showed proteinuria, microscopic haematuria, casts.

Subsequent testing revealed a marked elevation of aPS/PT IgM: 51.65 U/mL (normal <20; 2.58× ULN). This finding suggests a possible association between elevated aPS/PT IgM and the patient’s thrombotic phenotype, although contributions from other factors (e.g., family history of CAD, menopausal status) cannot be entirely excluded.

Based on arterial thrombosis (recurrent ISR), pathological pregnancy history, and positive aPS/PT IgM, the patient met the clinical definition of non−criteria APS. Treatment was intensified: warfarin (target INR 2.0–3.0), hydroxychloroquine (200 mg bid), plus continued DAPT.

**Figure 1:**
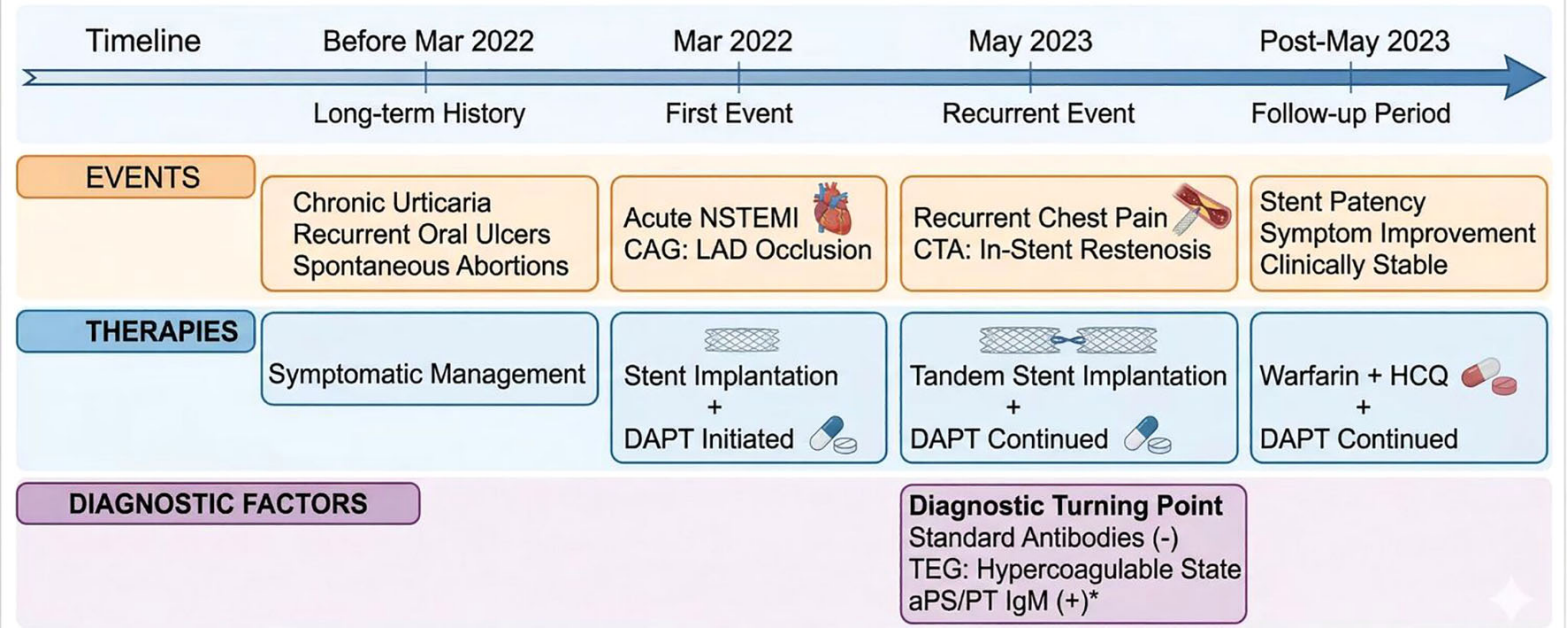
Diagnostic and therapeutic timeline.

**Figure 2:**
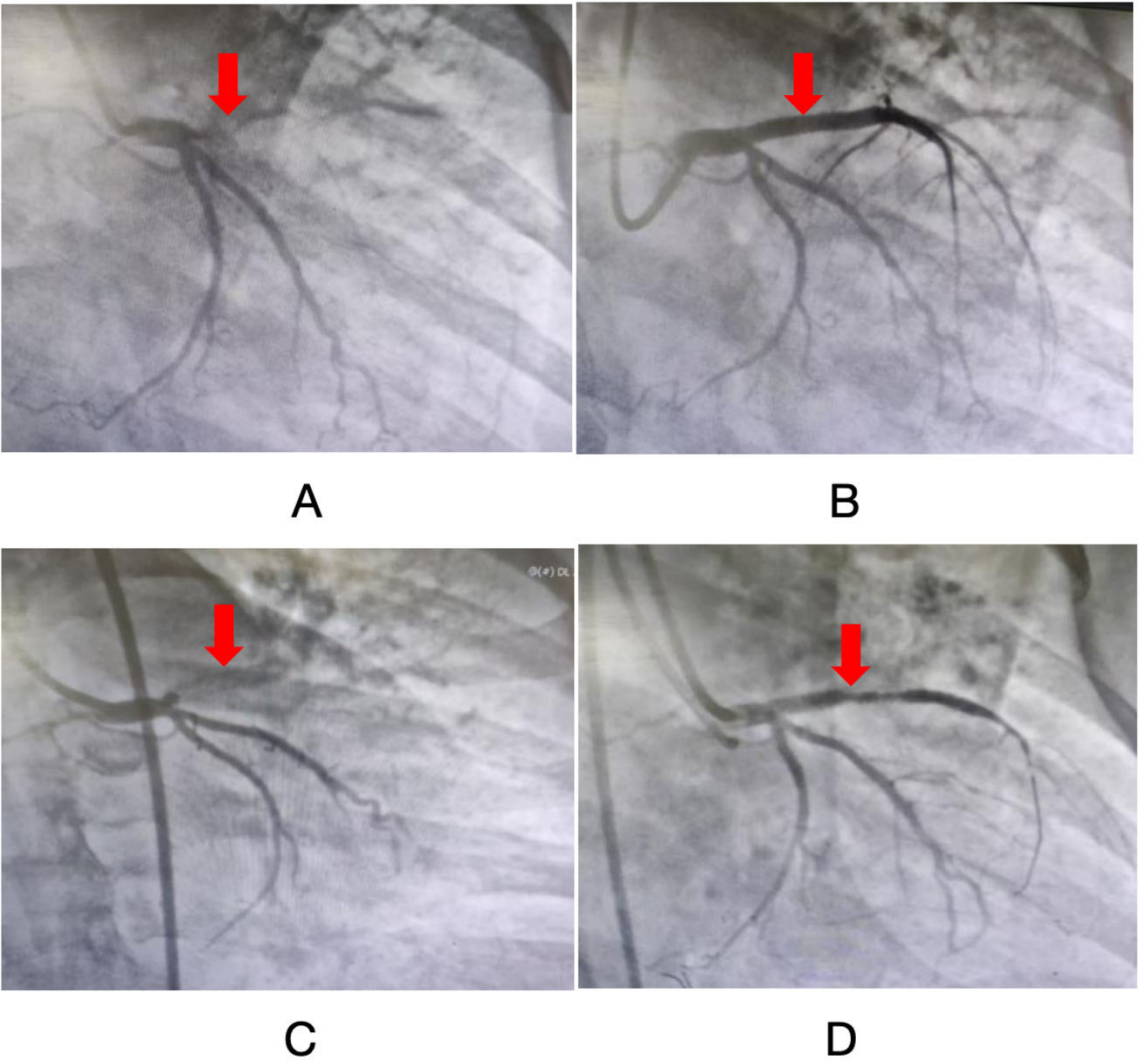
Coronary angiographic images. **(A)** Pre-first percutaneous coronary intervention (PCI), demonstrating total occlusion of the left anterior descending artery (LAD). **(B)** Post-first PCI with drug-eluting stent implantation in the LAD, showing restored luminal patency. **(C)** Pre-second PCI, revealing in-stent restenosis/occlusion of the LAD. **(D)** Post-second PCI with tandem stent implantation, confirming optimal stent apposition.

After initiation, INR remained therapeutic, D-dimer normalized, and repeat CTA confirmed stent patency. The patient adhered well to warfarin with no bleeding or adverse events. Chest pain did not recur; skin and oral lesions improved; anaemia resolved. During 21-month follow-up (June 2023 – March 2025), she remained clinically stable. The diagnostic and therapeutic timeline is illustrated in **Figure 1**, and the coronary angiographic images are shown in **Figure 2**.

## 3 Discussion

Unexplained recurrent arterial thrombosis in young patients without traditional risk factors should prompt evaluation for autoimmune disorders, including non−criteria APS. We report a case of recurrent in-stent restenosis ultimately attributed to non−criteria APS. Given the patient’s multisystem manifestations including oral ulcers, renal involvement, and skin lesions, other systemic autoimmune diseases, particularly systemic lupus erythematosus (SLE) and systemic vasculitis, were thoroughly evaluated and excluded. Laboratory examination showed positive ANA (1:80, speckled pattern) and elevated anti−SSA/Ro−60 and anti−SSA/Ro−52 antibodies, whereas all other key autoimmune markers including anti−dsDNA, ANCA, anti−MPO, anti−PR3, and complement C3/C4 were within normal limits or negative. These results indicate mild non−specific autoimmune activation rather than diagnostic features of SLE or other defined connective tissue diseases, and no clinical or serological features satisfied the classification criteria for SLE or systemic vasculitis. The absence of traditional risk factors and the failure of standard antiplatelet therapy to prevent rapid re-occlusion necessitated a search for an alternative, immune-mediated pathophysiology. This case suggests that unexplained recurrent stent failure may be a manifestation of underlying autoimmune thrombophilia, with aPS/PT IgM antibodies possibly playing a pathogenic role. The strength of this report is its comprehensive diagnostic journey and long-term follow-up, providing a practical roadmap for clinicians.

Classic antiphospholipid syndrome (APS) is defined by core phenotypes of thrombosis and adverse obstetric outcomes, coupled with persistent positivity for at least one standard antiphospholipid antibody in assays performed at least 12 weeks apart. The immunopathology of APS involves multiple interconnected pathways: autoantibodies bind to phospholipid-binding proteins on cell surfaces, leading to endothelial activation, upregulation of tissue factor, complement activation, and disruption of the annexin A5 anticoagulant shield (3, 7). APS patients are prone to microvascular thrombosis affecting multiple organs such as the skin, eyes, heart, lungs, and kidneys. Epidemiological data indicate a population prevalence of 40–50 per 100,000 individuals and an annual incidence of approximately 5 per 100,000 individuals (8, 9). In recent years, a subset of patients presenting with typical APS clinical features but persistently negative standard antibodies has been recognized and defined as non−criteria APS. Currently, several non-criteria antiphospholipid antibodies are used for adjunctive diagnosis, including anti-phosphatidylethanolamine (aPE) antibody, anti-β2-glycoprotein I domain I (aβ2GPI DI) antibody, aPS/PT antibody, and anti-vimentin/cardiolipin complex antibody (5, 10). Among these, aPS/PT antibody demonstrates a high positivity rate in thrombotic APS, and its titer correlates positively with thrombosis risk (11). It is also closely associated with fetal death (12), making it a core candidate marker for diagnosing non−criteria APS (4, 13). Mechanistically, aPS/PT antibodies may exert their prothrombotic effects through several immunological pathways: they can bind to prothrombin on endothelial cell surfaces, promoting local thrombin generation; they may activate complement via the classical pathway; and they can engage Fcγ receptors on immune cells, triggering inflammatory responses that predispose to thrombosis (14, 15). The diagnosis in this case was supported by the IgM titer (51.65 U/ml) which is moderately elevated (2.58×ULN; note that titers >80 U/mL are typically considered high by most kits). This suggests a potential contribution to the recurrent thrombotic event, although direct causality cannot be established from a single case.

Previous studies have shown that vascular involvement in APS can account for 2.8%-11% of cases, with aCL and aβ2GP1 antibodies considered key drivers of atherosclerosis and important non-traditional cardiovascular risk factors (9). However, our patient tested negative for standard antiphospholipid antibodies, and routine thrombophilia workup ruled out hereditary causes. Therefore, the recurrent thrombosis in this case is strongly attributed to aPS/PT antibody involvement. Notably, TEG provided objective functional evidence of a significant hypercoagulable state, supporting the presence of an acquired thrombophilia. TEG findings of decreased R time (shortened clotting time) and increased MA (maximum amplitude, reflecting maximal clot strength) and CI (coagulation index) are consistent with the hypercoagulable phenotype seen in autoimmune-mediated thrombosis (16). However, the utility of TEG in autoimmune disease remains investigational, and our finding should be interpreted cautiously. In contrast to aPS/PT IgG, which exhibits a predilection for venous thrombosis, some studies report no statistically significant difference in the association between aPS/PT IgM and arterial versus venous thrombosis (11, 17). Nevertheless, the moderately elevated titer of aPS/PT IgM in our patient may have induced both large-vessel (LAD occlusion and in-stent restenosis) and microvascular (recurrent abortion, renal endothelial injury, cutaneous microthrombosis) thrombotic events via potent endothelial activation and prothrombin-mimetic effects (14, 15, 18, 19). This broad spectrum of vascular involvement highlights the systemic nature of the immune-mediated process and suggests that aPS/PT IgM may target endothelial cells across different vascular beds. This may help explain the patient’s unique phenotype combining arterial thrombosis with multi-system involvement and offers clinical insights into the mechanisms of this antibody subtype (12).

Multiple case reports and series have linked aPS/PT antibodies to arterial thrombosis, including coronary events (11, 17). While the optimal treatment for non−criteria APS with arterial thrombosis remains undefined, anticoagulation is generally recommended based on evidence from classic APS (13).

The successful outcome in our patient is consistent with the use of anticoagulation in APS-related arterial thrombosis. Consistent with prior literature (20), patients with APS and CAD have a very high risk of recurrent thrombosis after intervention, necessitating intensive peri-procedural management. In this case, the addition of warfarin anticoagulation (target INR 2.0–3.0) after initial DAPT failure may have contributed to preventing further thrombotic events (13). The rationale for combining hydroxychloroquine lies in its dual role: first, as an immunomodulator, it may suppress the production of aPS/PT IgM at its source by interfering with Toll-like receptor signaling and antigen presentation in immune cells; second, by inhibiting platelet aggregation and protecting the vascular endothelium, it exerts an antithrombotic effect synergistic with warfarin. The combination of warfarin and hydroxychloroquine has been supported by series and some randomized controlled trials in APS (13, 21). This combination approach targets both the effector phase (thrombosis) and the induction phase (autoantibody production) of the autoimmune process, which likely contributed to the sustained clinical remission. For patients with a history of recurrent arterial thrombosis, as in our case, vitamin K antagonists remain the evidence-based cornerstone, although the use of direct oral anticoagulants is under investigation (13).

In summary, this case highlights a potential association between aPS/PT IgM and recurrent in-stent restenosis in a patient without traditional risk factors. The multisystem involvement (obstetric, renal, cutaneous) suggests a systemic autoimmune process. The significance of this study is that it draws attention to the possible role of non−criteria antibodies in unexplained coronary stent failure. Based on the aforementioned evidence, we suggest that testing for non−criteria antibodies such as aPS/PT may be considered as a potential diagnostic approach in patients with unexplained recurrent ISR and negative standard antiphospholipid (aPL) antibodies (5, 10, 17, 22).

## 4 Limitations

This study has several limitations. First, as a single case report, generalizability is limited. Second, due to real-world clinical constraints, we could not perform serial aPS/PT IgM monitoring; consequently, we cannot establish a quantitative relationship between titre fluctuations and thrombotic risk or treatment response, limiting our ability to define optimal thresholds for therapeutic adjustment. Furthermore, we did not repeat aPS/PT IgM testing at 12 weeks to confirm persistence, as required by classification criteria. Third, although we hypothesize specific immunological mechanisms (complement activation, Fc receptor engagement, endothelial activation), we did not perform complement assays or endothelial activation markers, which would have strengthened the mechanistic evidence. Fourth, LA testing was performed using only one method (diluted Russell viper venom time, dRVVT) instead of the recommended two methods; this is a limitation. TEG findings should be interpreted cautiously given the limited validation of this technique in autoimmune disease. Finally, the relatively short follow-up (21 months) may not capture late recurrences, which have been reported up to 5–10 years in APS.

## 5 Conclusion

Recurrent coronary in−stent restenosis in the absence of traditional risk factors should raise suspicion for non−criteria APS. A moderately elevated aPS/PT IgM may serve as a diagnostic clue for this arterial thrombotic phenotype. This case suggests that aPS/PT IgM could contribute to immune−mediated thrombosis even without classical antibodies. An integrated strategy of warfarin anticoagulation plus hydroxychloroquine immunomodulation may help prevent further thrombotic events. Rheumatologists, immunologists, and cardiologists should be aware of this possible association in patients with unexplained recurrent stent failure.

## 6 Patient perspective

The patient described her experience as follows: “The repeated chest pain and the need for two stent procedures in just over a year were frightening. I had also suffered multiple miscarriages over the years, which we never understood. After the second stent, when the doctors discovered this antibody and started me on blood thinners and hydroxychloroquine, my chest pain completely went away. My skin rashes and mouth ulcers, which I had for decades, also disappeared. For the first time, I feel like the underlying problem is being treated, not just the symptoms.”

## Data Availability

The original contributions presented in the study are included in the article/**Supplementary Material**. Further inquiries can be directed to the corresponding authors.

## Supplementary material

Supplementary Table 1 Laboratory examination results.
