# Supplementary material for: Case Report: Recurrent coronary in-stent restenosis as the primary manifestation of non−criteria antiphospholipid syndrome confirmed by anti-phosphatidylserine/prothrombin IgM antibodies
Source: Front Immunol. 2026 Jun 10;17:1852153. doi: 10.3389/fimmu.2026.1852153 (PMC13290989; doi:10.3389/fimmu.2026.1852153)
Supplement: Supplementary Table 1 — Laboratory examination results. [file DataSheet1.docx]

**Supplementary Table .** Laboratory Examination Results

| Examination Items | Results | Reference Range |
| --- | --- | --- |
| Anti-cyclic Citrullinated Peptide (anti-CCP) Antibody | 0.32 | Negative: <5.0 |
| Anti-PM-Scl Antibody IgG | 2.71 | Negative: <16.0 |
| Rheumatoid Factor (RF) IgG | 15.6 | Negative: <16.0 |
| Rheumatoid Factor (RF) IgM | 14.9 | Negative: <15.0 |
| Anticardiolipin (aCL) Antibody IgA | <2.5 | Negative: <8.0 |
| Anticardiolipin (aCL) Antibody IgM | <2.5 | Negative: <8.0 |
| Anticardiolipin (aCL) Antibody IgG | 6.67 | Negative: <8.0 |
| Anti-β2 Glycoprotein 1 (aβ2GP1) Antibody IgG | 4.93 | Negative: <16.0 |
| Anti-β2 Glycoprotein 1 (aβ2GP1) Antibody IgM | 0.59 | Negative: <16.0 |
| Antithrombin Ⅲ Activity (ATA) | 91 | 75-114 (%) |
| Protein S Activity (PSA) | 78 | 59-130 (%) |
| Protein C Activity (PCA) | 121.7 | 70-140 (%) |
| Lupus Anticoagulant (LA) Screen Test | 38.3 | 31-38 (s) |
| Lupus Anticoagulant (LA) Confirm Test | 34 | 30-38 (s) |
| Lupus Anticoagulant Ratio (LA1/LA1c) | 1.13 | 0.8-1.2 |
| Anti-double Stranded DNA (anti-dsDNA) Antibody Titer | Negative | Negative |
| Anti-neutrophil Cytoplasmic Antibody (ANCA) | Negative | Negative |
| Anti-granulocyte Cytoplasmic Antibody | Negative | Negative |
| Anti-myeloperoxidase (MPO) Antibody | Negative | Negative |
| Anti-proteinase 3 (PR3) Antibody | Negative | Negative |
| Total IgE | 286.1 | <100 (IU/mL) |
| High-sensitivity C-reactive Protein (hs-CRP) | 19.3↑ | 0-3 (mg/L) |
| Hemoglobin (Hb) | 83↓ | 115-150 (g/L) |
| Thromboelastography (TEG) |  |  |
| Reaction Time (R value) | 4↓ | 5-10 (min) |
| Maximum Amplitude (MA value) | 71.8↑ | 50-70 (mm) |
| Coagulation Index (CI value) | 3.2↑ | -3-3 |
